# Comprehensive Analysis of PDLIM3 Expression Profile, Prognostic Value, and Correlations with Immune Infiltrates in Gastric Cancer

**DOI:** 10.1155/2022/2039447

**Published:** 2022-05-12

**Authors:** Xinpeng Hu, Minfeng Chen, Qiang Ruan, Changzheng Shi, Jinghua Pan, Liangping Luo

**Affiliations:** ^1^Medical Imaging Center, The First Affiliated Hospital of Jinan University, Guangzhou 510630, China; ^2^College of Pharmacy, Jinan University, Guangzhou 510632, China; ^3^Department of The Second Area of Gastrointestinal Surgery, Affiliated Cancer Hospital and Institute of Guangzhou Medical University, Guangzhou 510095, China; ^4^Engineering Research Center of Medical Imaging Artificial Intelligence for Precision Diagnosis and Treatment, Guangzhou 510630, China; ^5^Department of General Surgery, The First Affiliated Hospital of Jinan University, Guangzhou 510630, China

## Abstract

Protein PDZ and LIM domain 3 (PDLIM3) is a cytoskeletal protein, colocalizing with *α*-actinin on the Z line of mature muscle fibers. It plays a key role in dilated cardiomyopathy (DCM), muscular dystrophy, and tumor progression. However, correlations between PDLIM3 expression, prognosis, and tumor-infiltrating immune cells in gastric cancer are unknown. Therefore, we leveraged the Oncomine, GEPIA, GEO, and HPA databases to evaluate PDLIM3 expression in tumors. We also quantified PDLIM3 expression in 15 matched pairs of gastric tumor and nontumor tissues by immunohistochemistry. The Kaplan-Meier method was employed to determine the relationship between PDLIM3 expression and clinical outcomes. GO and KEGG analyses were performed to illuminate the molecular mechanisms of action of PDLIM3. TIMER2.0 and GEPIA were applied to investigate correlations between PDLIM3 expression and gene marker subsets signifying immune infiltration, with TIMER2.0 exploring the correlations between PDLIM3 and related signaling pathways. Gastric cancer tissues were found to express more PDLIM3 than nontumor tissues. PDLIM3 overexpression was associated with shorter OS and PFS of gastric cancer patients (OS HR = 2.02, *P* = 9.8e − 10; PFS HR = 1.77, *P* = 7.5e − 06). PDLIM3 was also positively correlated with worse OS and PFS according to gastric cancer staging, Her-2 overexpression, differentiation grade, and Lauren classification. PDLIM3 was shown to be associated with immunological responses by GO, while it was related to PI3K/Akt signal pathways by KEGG analysis. Furthermore, increased PDLIM3 expression was significantly correlated with greater infiltration of CD4+ T cells, CD8+ T cells, macrophages, neutrophils, and dendritic cells. PDLIM3 expression had significant positive correlations with a variety of immune marker subsets. Finally, correlations were found between PDLIM3 and crucial markers of signaling pathways involving PI3K/Akt and p38 MAPK. Thus, upregulation of PDLIM3 was significantly associated with poor prognosis, immune cell infiltration, and activation of two key signal pathways in gastric cancer. We propose that PDLIM3 could be used as a biomarker to predict prognosis and immune cell infiltration in gastric cancer.

## 1. Introduction

Gastric cancer is becoming one of the most common cancers globally [1]. It conforms to a “three high and three low” pattern, i.e., high incidence, high metastasis rate, and high mortality and low early diagnosis rate, low radical resection rate, and low 5-year survival rate [2]. Gastric cancer is associated with chronic *H. pylori* infection, suppression of the T-helper 1 (Th1), and response and activation of CD8+ T cells, CD4+ T cells, as well as enhanced IFN production [3–5]. Immunotherapies deploying inhibitors of programmed death-1 (PD-1), programmed death ligand-1 (PD-L1), and cytotoxic T lymphocyte antigen 4 (CTLA-4) are important in the treatment of gastric cancer patients, especially in advanced cases, but unfortunately they have poor efficacy for this tumor type [6–8]. Notably, little is known in gastric cancer about tumor-associated macrophage (TAMs), tumor-infiltrating lymphocytes (TILs), and tumor-infiltrating neutrophils (TINs), which may all have a great impact on the efficacy of immunotherapy [6, 9]. Hence, more comprehensive knowledge of the mechanisms of immune modulation in gastric cancer is still required, and new targets for immune-related therapies are urgently needed.

Protein PDZ and LIM domain 3 (PDLIM3) is an actin-associated LIM protein (ALP) encoded on chromosome 4q35.1 [10, 11]. It colocalizes with *α*-actinin participating in a component of the Z line of mature muscle fibers, highly expressed in cardiac, smooth, and skeletal muscle [12]. PDLIM3 not only has an essential role in cellular differentiation, proliferation, and signal transduction but also participates in muscle stability, development, and cytoskeletal structural integration [13]. In addition, it was reported that PDLIM3 is linked to myotonic dystrophy, muscular dystrophy, and DCM [11, 14, 15]. Characterized by dissemination and metastasis, rhabdomyosarcoma has higher PDLIM3 levels in the bone marrow (BM) and peripheral blood (PB) than in normal BM and PB [16]. Thus, PDLIM3 might be a predictor of cancer progression, invasion, and metastasis.

A high level of expression of PDLIM3 was reported in medulloblastoma, bladder, and tongue carcinoma [17]. In medulloblastoma, high PDLIM3 gene expression was detected after hedgehog pathway activation, which could be an indication for using sonidegib treatment [18]. In another study, upregulated PDLIM3 expression was related to worse survival in bladder urothelial cancer and also correlated with EMT [19]. From the perspective of tumor immunity, EMT is related to the dampening of CD4+ and CD8+ T cell responses by overexpression of TGF-*β* and IL-10, as well as the upregulation of immune checkpoints such as CTLA-4 and TIM-3 in lung carcinoma [20]. In addition, PDLIM3 expression in breast carcinoma-associated fibroblasts (CAFs) was found to be significantly altered after treatment with Taxotere, which was closely related to chemotherapy resistance [21]. Myofibroblast products involve ALP, fibroblast activation protein, and myokine function as immunoregulators, modulating inflammation and immune cell trafficking [22–24]. For example, ALP member PDLIM2 inhibits T_H_17 cell development as well as granulomatous inflammation by targeting STAT3 [24]. The mechanisms responsible for PDLIM3 promotion of tumor progression and immune infiltration in gastric carcinoma are yet to be identified and are the subject of the present study.

## 2. Methods

### 2.1. Oncomine, Gene Expression Omnibus Database, and Human Protein Atlas Analysis

The Oncomine database is a cancer microarray database and integrated data-mining platform (https://www.oncomine.org/resource/login.html) [25]. PDLIM3 mRNA expression in different malignancies was interrogated in Oncomine. As a comprehensive gene expression library, the Gene Expression Omnibus (GEO) database is provided by the National Center of Biotechnology Information (NCBI) platform (https://www.ncbi.nlm.nih.gov/geo/). Finally, the Human Protein Atlas (HPA) may be used for determining protein expression in tumor samples and cell lines based on antibody immunostaining (https://www.Proteinatlas.org/) [26]. Here, we extracted PDLIM3 mRNA and protein expression data on gastric cancer and gastric nontumor tissues, analysed using the GEO database and HPA. Local Ethics Committee approval was not required for this part of the study because these are public databases.

### 2.2. Patients and Tissue Specimens

In the present study, 15 matched gastric tumor and adjacent nontumor tissues were investigated in patients undergoing radical surgical intervention at the Affiliated Cancer Hospital and Institute of Guangzhou Medical University between 1 January 2021 and 30 September 2021. All tissues were gathered after all patients and/or their guardians had signed informed consent. This part of the study was approved by the Ethics Board of the Affiliated Cancer Hospital and Institute of Guangzhou Medical University (No. P2019-022). All patients had stage II or III disease and underwent surgical resection, receiving no neoadjuvant chemotherapy or radiotherapy. Clinicopathological characteristics of the patients were retrospectively extracted from their medical records and pathology reports. Table S1 itemizes the clinical information of these 15 gastric cancer patients.

### 2.3. Immunohistochemistry

The 15 pairs of matched tissues were utilized for detecting PDLIM3 protein expression by immunohistochemistry (IHC), using an anti-PDLIM3 antibody purchased from Bioss (Beijing, China; bs-2928R). PDLIM3 immunostaining was detected as cytoplasmic staining under a light microscopy (Tokyo, Japan, BX53MRFS). For this, the average integral optical density (IOD) of five random fields was quantified at ×200 magnification.

### 2.4. Kaplan-Meier Plotter Database Analysis

The Kaplan-Meier Plotter database can be used to generate associations with survival of over 54000 genes across 21 malignancies (http://kmplot.com/analysis/) [27]. In the gastric cancer dataset, 631 and 511 samples were selected to investigate the impact of PDLIM3 mRNA expression on overall survival (OS) and progression-free survival (PFS). Additionally, this tool automatically computes log-rank *P* values and hazard ratios (HR) and generates survival plots.

### 2.5. PrognoScan Database Analysis

The Prognoscan database permits users to compare relationships between the expression of different genes and patients' outcomes for multiple cancer microarray datasets (http://www.abren.net/ PrognoScan/) [28]. Here, we assessed associations between PDLIM3 and outcomes of patients with blood, bladder, colorectal, breast, head and neck, eye, ovarian, lung, skin, and soft tissue cancers using PrognoScan.

### 2.6. GO Function and KEGG Pathway Enrichment Analyses

Gene Ontology (GO) enrichment results for biological process (BP), obtained by the R package “clusterprofiler”, were used to explore the functional roles of PDLIM3. Using the Kyoto Encyclopedia of Genes and Genomes (KEGG), pathways for PDLIM3-related enriched genes were generated by the R package.

### 2.7. TIMER2.0 Database Analysis

The TIMER2.0 database was developed to study immune cell infiltrates across various different malignancies (http://timer.cistrome.org/) [29]. Here, the PDLIM3 profile in gastric cancer patients was investigated and relationships with the abundance of immune cells infiltrating the tumor corrected for tumor purity. We aimed to determine the correlations between PDLIM3 level and infiltration of immune cells such as neutrophils, macrophages, dendritic cells, B cells, CD4+ T cells, and CD8+ T cells in patients with gastric cancer. The relations between PDLIM3 level and relevant immunological markers were assessed using correlation modules. Moreover, we explored correlations between PDLIM3 level and tumor-related signal pathways. The correlation module in TIMER2.0 was also used to evaluate the relationships between PDLIM3 expression and important molecules in crucial pathways. This correlation module used Spearman's correlation analysis to process coexpression data and generate scatter plots using correlation ratios and *P* values. The *x*-axis represents PDLIM3, whereas the *y*-axis represents the other desired genes.

### 2.8. GEPIA Database Analysis

The Gene Expression Profiling Interaction Analysis (GEPIA) database is an integrated gene expression analysis dataset that can be utilized to investigate 8,587 nontumor samples and 9,736 tumor samples from the GTEx and TCGA projects (http://gepia.cancer-pku.cn/index.html) [30]. We used GEPIA to investigate PDLIM3 levels in multiple carcinomas to correlate PDLIM3 expression with macrophage-, Treg-, and dendritic cell-specific markers in TIMER 2.0 for gastric cancer. Spearman's correlation analysis was conducted to determine correlation coefficients within a given expression dataset. Both tumor and nontumor sample datasets were leveraged for this analysis.

### 2.9. Statistical Analysis

*P* values, ranks, and fold-changes are included in the results from Oncomine. The paired *t*-test and Mann–Whitney *U* test were utilized to compare the PDLIM3 levels in gastric carcinoma and matched normal tissues and in the different pathological stages. GRAPHPAD PRISM 7 software was used to create graphical representations. Survival plots utilizing log-rank testing were generated by the Kaplan-Meier method. We used Spearman's correlation analysis to assess relationships between PDLIM3 and specific variables. The strength of a correlation was measured via *R* values and defined a very weak, 0.00–0.19; weak, 0.20–0.39; moderate, 0.40–0.59; strong, 0.60–0.79; and very strong, 0.80–1.0. *P* value < 0.05 was taken as statistically significant.

## 3. Results

### 3.1. Profiles of PDLIM3 Expression in Pan-Cancer

**Figure 1:**
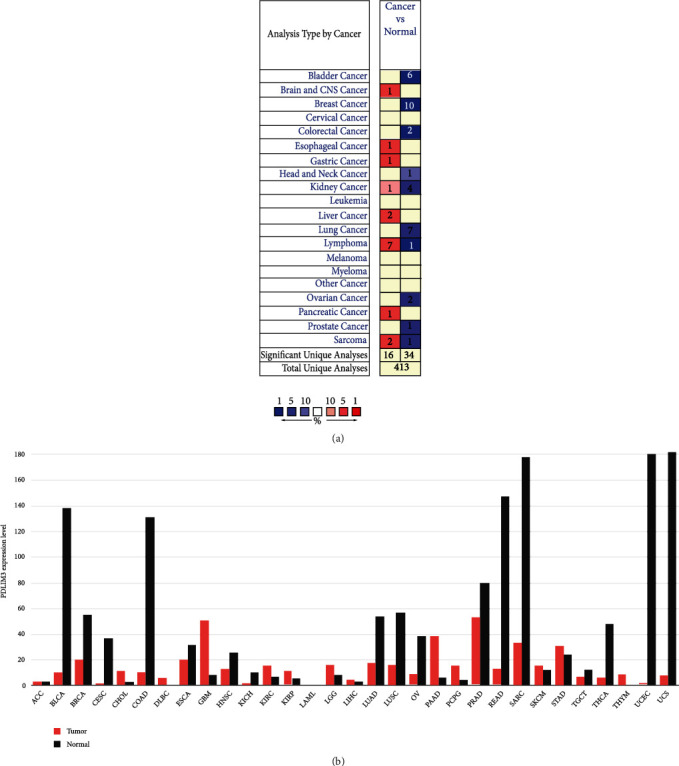
PDLIM3 expression profile in multiple types of human malignancies: (a) PDLIM3 in different malignancies compared to nontumor tissues in Oncomine database; (b) PDLIM3 mRNA level in multiple carcinomas types from GEPIA database.

PDLIM3 expression profiles varied in different malignancies, according to the Oncomine and GEPIA database results. We first compared PDLIM3 expression in different carcinomas and nontumor tissues via Oncomine data, finding that relative to nontumor tissues, PDLIM3 expression was elevated in esophageal, brain and CNS, kidney, gastric, pancreatic, liver cancer, lymphoma, and sarcoma (Figure 1(a)). However, breast, bladder, head and neck, colorectal, lung, kidney, prostate, ovarian cancer, lymphoma, and sarcoma all exhibited lower expression. Oncomine data were also utilized to summarize the expression of PDLIM3 in several types of cancers (for detailed results, see Table S2). Moreover, the expression of PDLIM3 in multiple carcinomas from the TCGA database was summarized using the GEPIA database (Figure 1(b)).

### 3.2. Upregulated PDLIM3 in Gastric Cancer

**Figure 2:**
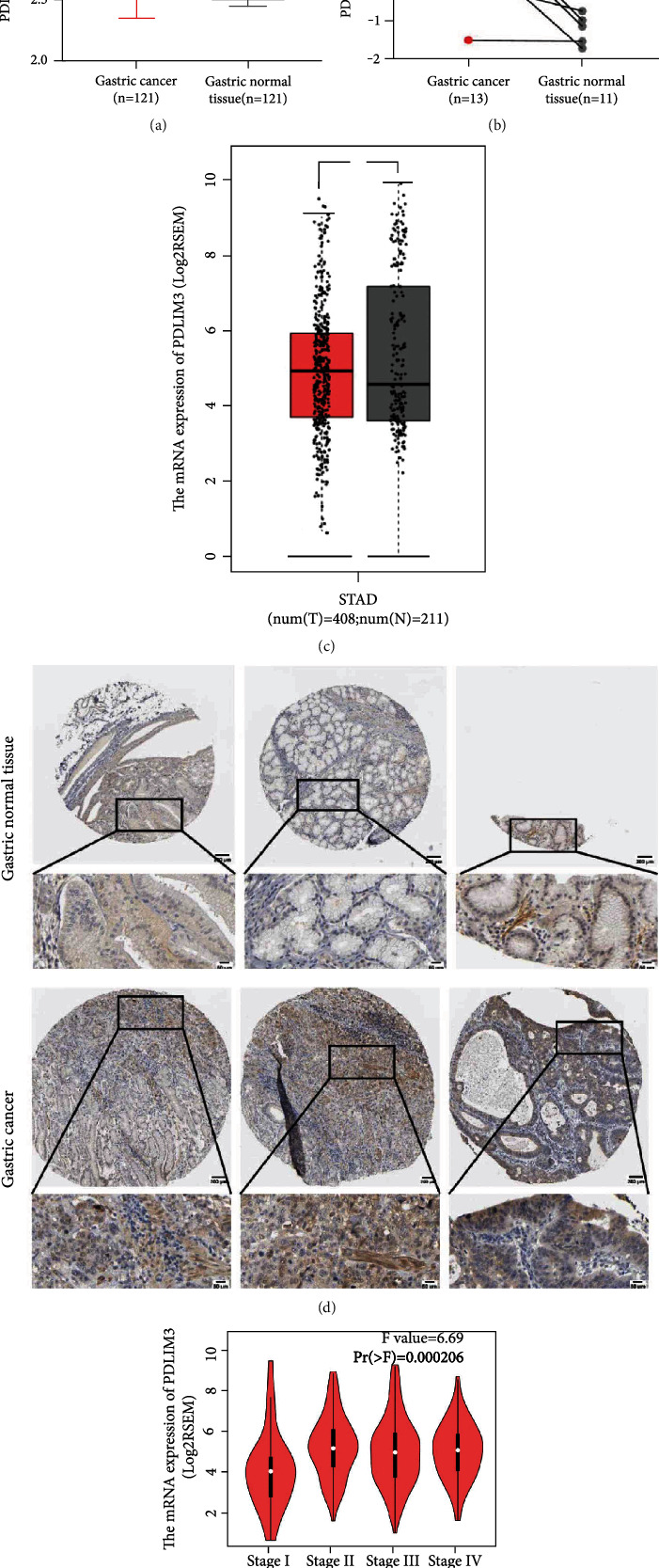
PDLIM3 mRNA and protein expression in gastric cancer. (a and b) PDLIM3 mRNA level in the GSE54129 and GSE118916 datasets. (c) PDLIM3 mRNA profile in GEPIA. (d) PDLIM3 protein profile in HPA database. PDLIM3 protein staining was negative in nontumor tissues (gastric normal tissue) and moderate in tumor tissues (gastric cancer). (e) PDLIM3 mRNA profile in pathological stages.

**Figure 3:**
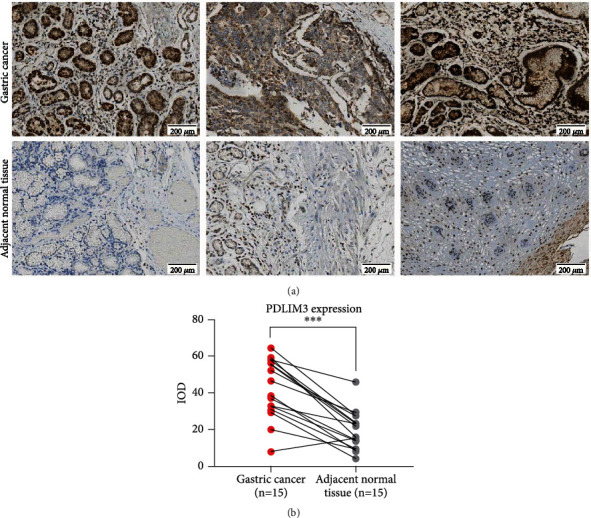
Comparison of PDLIM3 IHC staining between 15 matched tumor tissues (gastric cancer) and nontumor tissues (adjacent normal tissue): (a) IHC staining results of PDLIM3; (b) Analysis of PDLIM3 IHC results.

Based on the data from the GEO, GEPIA, and HPA databases, higher PDLIM3 mRNA and protein levels were present in gastric carcinoma than in matched control tissues. Significant upregulation of PDLIM3 mRNA was observed in GSE54129 and GSE118916 in GEO (both with *P* < 0.001) (Figures 2(a) and 2(b)). Also in the GEPIA gastric carcinoma tissues, PDLIM3 was shown to be elevated in tumors (*n* = 408) compared to nontumor tissues (*n* = 211) (*P* < 0.05, Figure 2(c)), with differences seen in different pathological stages (Pr = 0.000206, Figure 2(e)). In the HPA database, PDLIM3 protein expression and antibody staining levels in gastric tumors were moderate, while nontumor tissues were negative (Figure 2(d)). To further confirm these findings, IHC staining was used to analyse the PDLIM3 protein profile in 15 matched pairs of gastric tumor and nontumor tissues (Figures 3(a) and 3(b). As expected, the results revealed that the average IOD of PDLIM3 immunostaining in gastric tumors (41.7 ± 16.5) was significantly stronger than in matched nontumor samples (19.5 ± 10.8) (*P* < 0.001).

### 3.3. Potential Diagnostic Value of PDLIM3 in Gastric Cancer

**Figure 4:**
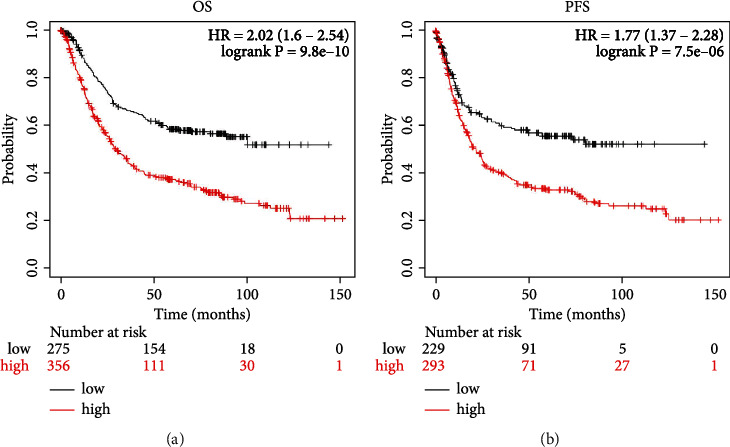
The Kaplan-Meier survival curves of comparing discrepant expression of PDLIM3 in gastric cancer: (a) Overall survival (OS) curves; (b) Progression-free survival (PFS) curves.

To investigate the prognostic value of PDLIM3 in gastric carcinomas, the Kaplan-Meier Plotter analysis was conducted to generate survival plots. High PDLIM3 expression was found to be correlated with a lower overall survival time in patients with gastric carcinoma (HR = 2.02, 95% CI = 1.6 to 2.54, Cox *P* = 9.8e − 10, Figure 4(a)). High PDLIM3 expression was similarly associated with considerably worse outcomes also for PFS (HR = 1.77, 95%CI = 1.37 to 2.28, Cox *P* = 7.5e − 06, Figure 4(b)). We then applied Prognoscan database analysis to further investigate the influence of PDLIM3 level on survival rates, and to evaluate the correlation of PDLIM3 with the prognosis of patients with different malignancies (Table S3). We found a clear association between PDLIM3 and prognosis of blood, breast, colorectal, ovarian, skin, and soft tissue cancer patients.

### 3.4. Impact of PDLIM3 Expression on Clinicopathological Characteristic of Gastric Cancer

To further explore the impact of high PDLIM3 expression on survival, we analysed correlations between PDLIM3 mRNA levels and clinicopathological characteristics of patients with gastric carcinoma. We found that upregulation of PDLIM3 was associated with worse OS of patients at stage 1, 3, and 4; stage T2 to T4; stage N0 to N3; and stage M0, 1 and also worse PFS at stage 3; stage T2 to T4; stage N0 to N3; and stage M0, 1 (Table 1). Increased PDLIM3 expression was associated with poorer OS and PFS according to sex, Her-2 status, moderate differentiation, and the Lauren classification (*P* < 0.05), but was not associated with PFS of mixed type disease (PFS HR = 1.76, 95%CI = 0.61 to 5.1, Cox *P* = 0.2907). In addition, increased PDLIM3 expression showed the highest association of with OS and PFS in N1 + 2 + 3. Taken together, these results further confirm that increased PDLIM3 expression is a predictive indicator of mortality risk in gastric cancer patients, especially when lymph node metastases are present.

### 3.5. Functional Annotation and Pathway Enrichment of PDLIM3-Related Genes in Gastric Cancer

**Figure 5:**
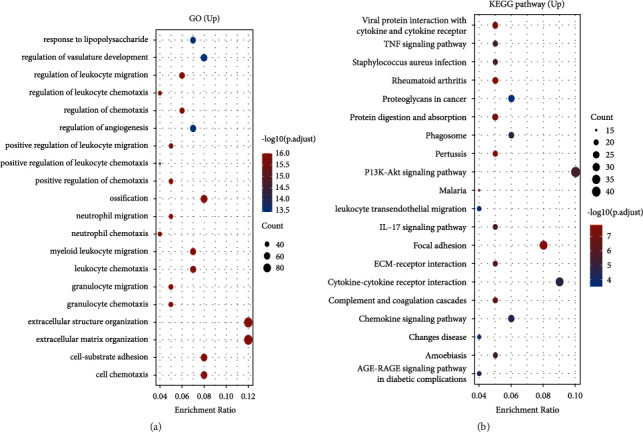
PDLIM3 gene enrichment analysis of GEO dataset: (a) Enriched GO terms of PDLIM3-related genes in GSE54129; (b) Enriched KEGG pathways of PDLIM3-related genes in GSE54129.

To further investigate the cellular functions and molecular mechanisms of PDLIM3 action in gastric cancer, PDLIM3-related genes identified in GSE54129 of GEO were used to perform GO and KEGG analyses. The top twenty enriched BP terms were mainly included in the regulation of immune responses, followed by extracellular matrix formation (Figure 5(a)). Moreover, KEGG pathway enrichment analysis revealed that PDLIM3-related genes were primarily enriched in the PI3K/Akt signaling pathway, related to oncogenesis (Figure 5(b)). Leukocyte transendothelial migration and ECM-receptor interaction pathways were also found to be involved. These findings imply that PDLIM3 has a crucial role in PI3K/Akt-related tumorigenesis, immune responses, and extracellular matrix formation.

### 3.6. Relationships between PDLIM3 and Immune Infiltration in Gastric Cancer

**Figure 6:**
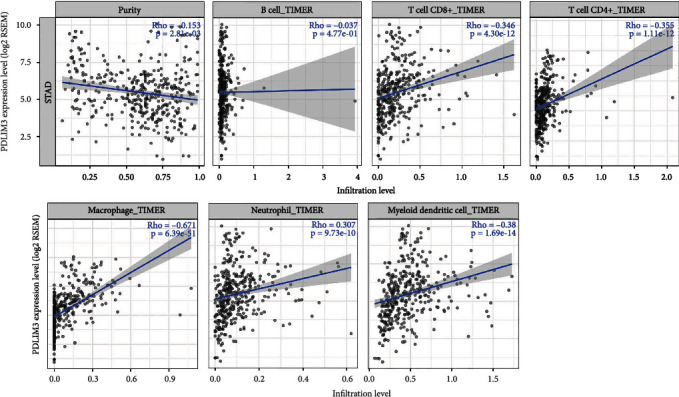
Correlation of PDLIM3 with immune infiltration level in gastric cancer. PDLIM3 expression is related to infiltrating levels of CD4 + T cells, CD8 + T cells, macrophages, neutrophils, and dendritic cells.

Because of different degrees of immune cell infiltration, patients with the same histological types of cancer may have different clinical outcomes [31]. Tumor-infiltrating lymphocytes have been found to be a specific predictor of sentinel lymph node conditions and outcome in cancer patients [32, 33]. Based on the results from the GO analysis, we utilized the TIMER2.0 database to explore whether PDLIM3 levels were associated with immune cell infiltrates in gastric carcinoma. We found that PDLIM3 expression was positively correlated with immune cell infiltration, while it was negatively correlated with gastric cancer tumor purity (Figure 6). These results indicate that high PDLIM3 expression was most significantly correlated with macrophages (Cor = 0.671, *P* = 6.39e − 51), followed by myeloid dendritic cells (Cor = 0.38, *P* = 1.69e − 14), CD4+ T cells (Cor = 0.355, *P* = 1.11e − 12), CD8+ T cells (Cor = 0.346, *P* = 4.30e − 12), and neutrophils (Cor = 0.307, *P* = 9.73e − 10). In contrast, high PDLIM3 expression was only very weakly correlated with the infiltration of B cells (Cor = 0.037, *P* = 4.77e − 1). These findings may provide an explanation for why PDLIM3 has a positive association with immune cell infiltration in gastric cancer, especially with macrophages.

### 3.7. Correlations between PDLIM3 and Marker Subsets of Immune Cells

**Figure 7:**
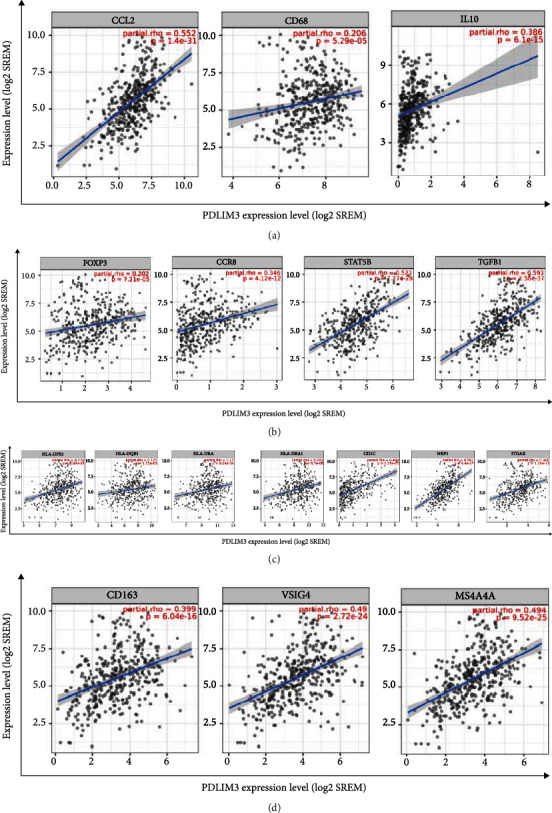
Correlation between PDLIM3 expression and biomarkers of macrophage, Treg, and dendritic cell in gastric cancer: (a) CCL2, CD68, and IL10 of TAM; (b) FOXP3, CCR8, STAT5B, and TGF*β* (TGFB1) of Treg; (c) HLA-DPB1, HLA-DQB1, HLA-DRA, HLA-DPA1, BDCA-1 (CD1C), BDCA-4 (NRP1), and CD11c (ITGAX) of dendritic cell; (d) CD163, VSIG4, and MS4A4A of M2 macrophage.

To explore correlations between levels of PDLIM3 expression and different immune cells infiltrating gastric carcinoma, we used the TIMER2.0 database to investigate correlations between PDLIM3 and marker subsets of immune cells including B cells, T cells, macrophages, monocytes, neutrophils, natural killer, and dendritic cells. After adjusting by purity, PDLIM3 expression was positively correlated with a majority of marker subsets of T cells (CD8+, general T cells, Th1, Th2, Tfh, Th17, Tregs, and exhausted), B cells, monocytes, macrophages (M1, M2, and tumor-associated), neutrophils, natural killer (NK), and dendritic cells (Table 2). In addition, moderately positive correlations with PDLIM3 expression were found for markers of TAMs, M2 macrophages, Tregs, and dendritic cells. Marked positive correlations were found between PDLIM3 and CCL2 expression by TAMs (*R* = 0.552, *P* = 1.4e − 31), VSIG4 (*R* = 0.49, *P* = 2.72e − 24), and MS4A4A (*R* = 0.494, *P* = 9.52e − 25) by M2 macrophages; STAT5B (*R* = 0.522, *P* = 7.27e − 28) and TGF*β* (*R* = 0.593, *P* = 2.56e − 37) by Tregs; and BDCA-1 (*R* = 0.483, *P* = 1.25e − 23) and BDCA-4 (*R* = 0.591, *P* = 4.4e − 37) by dendritic cells (Figures 7(a)–7(d)). To further confirm the substantial connections between PDLIM3 expression level and TAMs, M2 macrophages, Tregs, and dendritic cells, we accessed the GEPIA database. Correlations between PDLIM3 expression and the above markers in GEPIA (Table 3) were similar to those found in TIMER2.0. Thus, these results revealed that the PDLIM3 level is closely associated with the immune cells infiltrating gastric cancer, implying that it acts as a crucial factor of immune escape in the gastric cancer microenvironment.

### 3.8. Correlation of PDLIM3 Expression with PI3K/Akt and p38 MAPK Signaling Pathways

**Figure 8:**
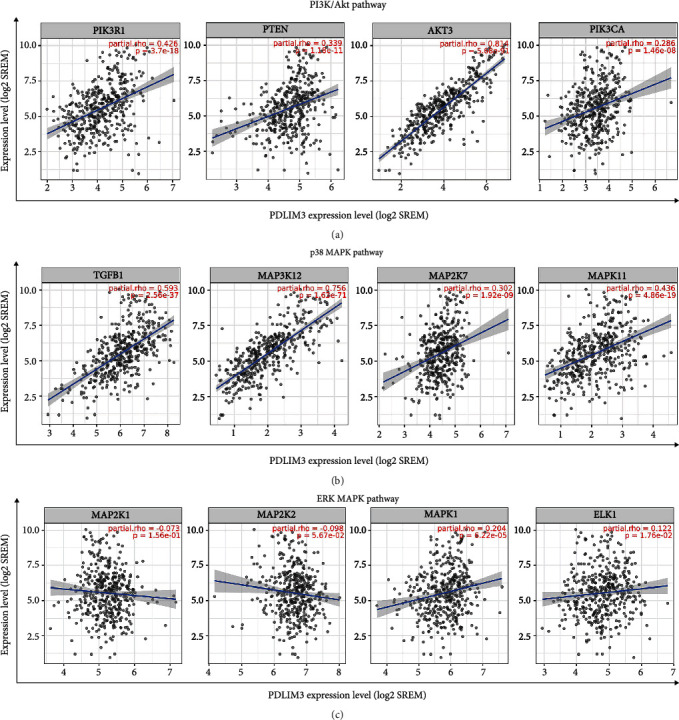
Correlation of PDLIM3 expression with biomarkers of enriched KEGG pathways in gastric cancer: (a) PI3K (PIK3R1), PTEN, PKB-*γ*(AKT3), and BRAF (PIK3CA) of PI3K/Akt pathway; (b) TGF-*β* (TGFB1), DLK (MAP3K12), MEK7 (MAP2K7), and P38*β* (MAPK11) of p38 MAPK pathway; (c) MEK1 (MAP2K1), MEK2 (MAP2K2), ERK1 (MAPK1), and ELK1 of ERK MAPK pathway.

Based on the results of the KEGG analysis, we sought correlations between the PDLIM3 level and activation status of the PI3K/Akt pathway, well known for its important impact on the development of gastric carcinoma. The PDLIM3 profile was found to be positively correlated with the expression of the genes for PI3K (PIK3R1) (*R* = 0.426, *P* = 3.7e − 18), PTEN (*R* = 0.339, *P* = 1.16e − 11), PKB-*γ*(AKT3) (*R* = 0.814, *P* = 15.88e − 91), and BRAF (PIK3CA) (*R* = 0.286, *P* = 3.7e − 18) (Figure 8(a)). Based on the correlation between PDLIM3 and BRAF, a MAPKKK in the MAPK pathway, we investigated the correlations between PDLIM3 expression and activation states of p38 MAPK and ERK MAPK pathways. The PDLIM3 profile was found to be significantly positively correlated with the level of expression of TGF-*β* (TGFB1) (*R* = 0.593, *P* = 2.56e − 37), DLK (MAP3K12) (*R* = 0.756, *P* = 1.6e − 71), MEK7 (MAP2K7) (*R* = 0.302, *P* = 1.92e − 9), and P38*β* (MAPK11) (*R* = 0.436, *P* = 4.86e − 19) in the p38 MAPK pathway (Figure 8(b)). However, only weak correlations were found between PDLIM3 and the ERK MAPK pathway involving MEK1 (MAP2K1) (*R* = −0.073, *P* = 1.56e − 1), MEK2 (MAP2K2) (*R* = −0.098, *P* = 5.67e − 2), ERK1 (MAPK1) (*R* = 0.204, *P* = 6.22e − 5), and ELK1 (*R* = 0.122, *P* = 1.76e − 2) (Figure 8(c)).

## 4. Discussion

Gastric cancer is a common malignancy with a high disease-specific mortality rate. In spite of utilizing a variety of therapeutic options, gastric cancer patients are still suffering from unsatisfactory efficacies and high mortality rates. PDLIM3, a member of the ALP family, contributes to the formation of the Z line in mature muscle fibers and functions as a modulator of muscle cell activities such as cell differentiation, proliferation, signal transduction, stability, development, and cytoskeletal structural integration [13]. Previous research had revealed that PDLIM3 had carcinogenic potential, with higher expression levels being associated with a variety of malignancies and being relevant to poorer survival, chemotherapy resistance, and EMT [17–20]. Another ALP family member, PDLIM2, could mediate granulomatous inflammation by suppressing the growth of TH17 cells [24]. Even so, the relationship between PDLIM3 and the progression and immunology of tumors remained unknown. Based on these data, we investigated the levels of PDLIM3 in gastric carcinoma via several database analyses and IHC. As a result, we found that PDLIM3 expression was upgraded in gastric cancer, especially later stages. The prognoses of gastric cancer patients were negatively impacted by high PDLIM3 expression, most relevant at later cancer stages, with the highest HR value identified in patients with N1 + 2 + 3 disease. Moreover, we found that PDLIM3-related genes were enriched for regulation of immune responses and tumorigenesis-related PI3K/Akt pathway activation. PDLIM3 was significantly correlated with both immune cell infiltrates and immune-specific marker subsets and two important pathways in gastric cancer. Therefore, PDLIM3 acts as a potential biomarker and participant in tumor immunity in gastric carcinoma.

The transcriptional and translational status of PLDIM3 expression was examined in gastric cancer using the Oncomine, GEO, GEPIA, and HPA databases. We identified significant differences between tumor and nontumor samples in gastric cancer. First, the Oncomine database analysis revealed that higher PDLIM3 mRNA expression was found in esophageal, brain and CNS, kidney, gastric, pancreatic, liver cancer, lymphoma, and sarcoma, while breast, bladder, head and neck, colorectal, lung, kidney, prostate, ovarian cancer, lymphoma, and sarcoma exhibited lower levels. Second, PDLIM3 expression in multiple malignancies from the TCGA database was also investigated using the GEPIA database. In GEO (GSE54129, GSE118916) and the GEPIA database, we found that PDLIM3 mRNA was increased in gastric cancer tissues and later pathological stages. Third, the HPA database analysis revealed that PDLIM3 expression at the protein levels was higher in gastric carcinoma than in nontumor tissues. In order to further validate this result, PDLIM3 expression was shown to be higher in gastric carcinoma than in matched nontumor samples of 15 paired clinical specimens by IHC.

In one part of this study, we found that upregulation of PDLIM3 expression predicted worse prognosis in association with numerous clinicopathologic features in gastric cancer. According to the Kaplan-Meier Plotter database analysis, elevated PDLIM3 was associated with a worse prognosis for both OS and PFS. High levels of PDLIM3 were also found in various malignancies involving breast, blood, ovarian, colorectal, skin, and soft tissue cancer associated with a worse prognosis. Furthermore, upregulation of PDLIM3 predicted worse OS and PFS in stage 3, stage T2 to T4, stage N0 to N3, and stage M0, but worse OS not PFS in stage 2 and 4. The highest HR value was identified in N1 + 2 + 3 disease. Higher levels of PDLIM3 expression, which were paralleled by Her-2 overexpression, differentiation, and the Lauren classification, also influenced the prognosis of gastric cancer patients.

In another part of the study, we investigated the cellular functions and molecular mechanisms of PDLIM3 in gastric carcinoma, using GO and KEGG analyses. The GO categories were mainly enriched in the modulation of immune responses, followed by extracellular matrix formation. KEGG pathway analysis demonstrated that PDLIM3-related genes were primarily enriched in the PI3K/Akt pathway, associated with oncogenesis. A recent study showed that TILs were closely correlated with clinical prognosis in gastric cancer [34]. To more deeply explore the correlation between PDLIM3 and tumor-related immune responses in gastric cancer, we found that the expression of this molecule was quantitatively associated with the degree of immune infiltration and specific immunological markers. In the present study, PDLIM3 was positively associated with infiltration of CD4+ T cells, CD8+ T cells, macrophages, neutrophils, and dendritic cells. Macrophages including TAMs, showing the highest correlation with PDLIM3, play a comparatively important role not only in progression, metastasis, and angiogenesis of tumors but also in immune suppression and therapy resistance [35, 36]. If activated appropriately, macrophages play a role in antitumoral action via cooperating with T cells as well [37]. This finding reveals a potential correlation between PDLIM3 and tumor macrophage infiltration. More clearly demonstrating its potential to regulate tumor immunity, marked positive correlations were found between PDLIM3 and specific immunological markers. First, moderately positive correlations were found with CCL2 in TAMs. Tumor cell migration is mediated by immune and tumor cell interactions via CCL2-CCR2, which might reflect the tumor-promoting effect of PDLIM3 in facilitating the infiltration of TAMs into gastric cancer [38, 39]. Second, upregulated PDLIM3 was moderately correlated with VSIG4 and MS4A4A in M2 macrophages, rather than M1 macrophages. Cancer development is known to be influenced by VSIG4 and MS4A4A, respectively, blocking T-cell activation and activating NK-cell-mediated resistance to metastasis [40, 41]. We surmise that PDLIM3 not only induces polarization of M2 macrophages but also participates in the regulation of tumorigenesis by T and NK cells. Third, our results showed that PDLIM3 expression was significantly positively correlated with STAT5B and TGF*β* in Tregs and BDCA-1 and BDCA-4 in dendritic cells. TGF*β*, expressed by Tregs, is an immune suppressor and fibroblast differentiation regulator [42, 43]. In animal models, the effect of anti-PD-1 checkpoint blockade required crosstalk between T cells and dendritic cells via the cytokines interleukin-12 (IL-12) and interferon-*γ* (IFN-*γ*) [44]. The high level of IL-12 and IFN-*γ* secretion by human BDCA-1^+^ dendritic cells indicates that PDLIM3 might participate in crosstalk between T cells and dendritic cells [45]. Thus, all these results imply that PDLIM3 is intimately associated with immune cell infiltration into gastric carcinoma, suggesting that it plays a crucial role in immunologic escape in the gastric cancer microenvironment.

Additionally, PDLIM3-related genes were also shown to be enriched in extracellular matrix formation, according to the GO and KEGG categories. In a recent study, PDLIM3 was recognized as a potential marker for tumor stroma in colorectal cancer CAFs and classified as one of the genes conferring a high risk of recurrence [46]. After treatment with Taxotere, PDLIM3 expression by breast CAFs was found to be significantly altered, which was associated with chemotherapy resistance [21]. We hypothesize that the PDLIM3 profile plays a role in CAF-mediated extracellular matrix formation in gastric cancer.

Based on PDLIM3-related enrichment of PI3K/Akt pathway genes, we explored the correlations between PDLIM3 and crucial markers of the PI3K/Akt pathway. We found that PDLIM3 expression was positively correlated with such markers including PI3K, PTEN, PKB-*γ*, and BRAF in gastric cancer. The PI3K/Akt pathway is involved in a wide range of diseases, cancer progression, and metastasis, including gastric cancer [47–49]. Considering that BRAF in PI3K/Akt is also a MAPKKK of MAPK pathway, a positive correlation between BRAF and PDLIM3 could imply MAPK pathway activity as well [50]. We therefore sought correlations between PDLIM3 and crucial markers of the p38 MAPK and ERK MAPK pathways in gastric cancer. The PDLIM3 profile was shown to be significantly positively correlated with such markers of the p38 MAPK pathway as TGF-*β*, DLK, MEK7, and P38*β*, but only weakly with the ERK MAPK-associated factors MEK1, MEK2, ERK1, and ELK1. Chicken muscle cell proliferation and differentiation were reported to be inhibited by decreasing PDLIM3 through the p38 MAPK pathway [13]. This p38 MAPK pathway also facilitates tumor formation and metastasis in several cancer types [51–53]. Moreover, the recruitment of macrophages and Tregs via CCL2 secretion could be suppressed by p38 inhibition in hepatocellular carcinoma, thus inhibiting tumor growth and metastasis [54]. Promoting tumor-stroma formation, gastric cancer exosomes triggered human umbilical cord-derived mesenchymal stem cell (hucMSC) differentiation into CAFs through p38 activation, which could be reversed by TGF-*β* inhibition [55]. Therefore, crosstalk between PDLIM3 and PI3K/Akt, p38 MAPK pathways may be the mechanism responsible for correlations between PDLIM3 and poorer prognosis and immune infiltration in gastric cancer.

There are limitations that consist in this article. First, PDLIM3 expression of clinical samples was analysed by IHC, and its prognostic significance needs to be further confirmed in clinical cases. Second, how PDLIM3 modulates immune infiltrates was explored with the public databases, and both in vivo and in vitro experiments need to be investigated.

## 5. Conclusions

In summary, elevated expression of PDLIM3 is present in gastric cancer, associated with poor prognosis, high immune cell infiltration, and signal pathway activation. The present study indicates that PDLIM3 is a predictive biomarker for immune infiltration in gastric cancer.

## Figures and Tables

**Table 1:** Correlation of PDLIM3 mRNA level and clinical outcomes in gastric cancer with multiple clinicopathological factors by the Kaplan-Meier plotter.

Clinicopathological characteristics	Overall survival (*n* = 881)			Progression-free survival (*n* = 645)		
	*N*	Hazard ratio	*P* value	*N*	Hazard ratio	*P* value
Gender						
Female	244	2.01 (1.48 − 2.72)	4.3e − 06	179	2.61.62 − 4.34)	0.003
Male	566	2.73 (1.77 − 4.22)	2.6e − 06	341	1.56 (1.15 − 2.12)	0.003
Stage						
1	69	4.21 (0.9 − 18.92)	0.0491	62	3.42 (074 − 15.72)	0.0939
2	145	1.87 (0.95 − 3.65)	0.0639	131	1.67 (0.9 − 3.09)	0.1012
3	319	2.72 (1.79 − 4.13)	1.1e-06	186	2.09 (1.41 − 3.11)	0.0002
4	152	2.04 (1.36 − 3.06)	0.0004	141	1.43 (0.97 − 2.11)	0.0686
Stage T						
2	253	1.59 (1.01 − 2.48)	0.0416	239	2.12 (1.4 − 3.21)	0.0003
3	208	2.01 (1.35 − 2.97)	0.0004	204	1.56 (1.12 − 2.18)	0.0088
4	39	3.97 (1.58 − 9.99)	0.0016	39	3.07 (1.24 − 7.58)	0.0108
Stage N						
0	76	6.74 (1.57 − 29.03)	0.0031	72	6.21 (1.45 − 26.7)	0.0051
1	232	2.55 (1.68 − 3.86)	0.000005	222	2.27 (1.47 − 3.52)	0.0002
2	129	2.27 (1.43 − 3.6)	0.0004	125	1.99 (1.27 − 3.1)	0.0021
3	76	2.21 (1.27 − 3.83)	0.0039	76	1.87 (1.08 − 3.22)	0.0229
1 + 2 + 3	437	2.07 (1.59 − 2.7)	3.1e − 08	423	1.74 (1.35 − 2.24)	1.5e-05
Stage M						
0	459	2.14 (1.58 − 2.9)	5.1e − 07	443	2.2 (1.4 − 3.47)	0.0005
1	58	2.8 (1.47 − 5.35)	0.0012	56	1.55 (0.81 − 2.95)	0.1796
Lauren classification						
Intestinal	336	2.36 (1.64 − 3.4)	0.000002	263	1.93 (1.36 − 2.75)	0.0002
Diffuse	248	2.11 (1.43 − 3.11)	0.0001	231	1.82 (1.24 − 2.66)	0.0018
Mixed	33	3.11 (1 − 9.69)	0.0402	28	1.76 (0.61 − 5.1)	0.2907
Differentiation						
Poor	166	0.77 (0.47 − 1.25)	0.2838	121	0.73 (0.42 − 1.25)	0.2449
Moderate	67	3.53 (1.24 − 10)	0.0115	67	4.01 (1.42 − 11.32)	0.0045
HER-2 status						
Negative	641	2.13 (1, 59 − 2.85)	1.8e − 07	25	1.88 (1.37 − 2.59)	0.00008
Positive	424	2.22 (1.48 − 3.34)	0.000079	42	2.14 (1.37 − 3.35)	0.0006

**Table 2:** Correlation analysis between PDLIM3 and related gene markers of immune cells in TIMER 2.0.

Description	Gene markers	STAD			
		None		Purity	
		Cor	*P*	Cor	*P*
CD8+ T cell	CD8A	0.31	^∗∗∗^	0.276	^∗∗∗^
	CD8B	0.17	^∗∗^	0.151	^∗^

T cell (general)	CD3D	0.246	^∗∗∗^	0.205	^∗∗∗^
	CD3E	0.267	^∗∗∗^	0.23	^∗∗∗^
	CD2	0.3	^∗∗∗^	0.265	^∗∗∗^

B cell	CD19	0.321	^∗∗∗^	0.306	^∗∗∗^
	CD79A	0.349	^∗∗∗^	0.317	^∗∗∗^

Monocyte	CD86	0.403	^∗∗∗^	0.376	^∗∗∗^
	CD115 (CSF1R)	0.508	^∗∗∗^	0.489	^∗∗∗^

TAM	CCL2	0.573	^∗∗∗^	0.552	^∗∗∗^
	CD68	0.228	^∗∗∗^	0.206	^∗∗∗^
	IL10	0.399	^∗∗∗^	0.386	^∗∗∗^

M1 macrophage	INOS (NOS2)	-0.055	2.65e − 01	-0.061	2.39e − 01
	IRF5	0.247	^∗∗∗^	0.239	^∗∗∗^
	COX2 (PTGS2)	0.232	^∗∗∗^	0.239	^∗∗∗^

M2 macrophage	CD163	0.417	^∗∗∗^	0.399	^∗∗∗^
	VSIG4	0.5	^∗∗∗^	0.49	^∗∗∗^
	MS4A4A	0.511	^∗∗∗^	0.494	^∗∗∗^

Neutrophils	CD66b (CEACAM8)	-0.01	8.34e − 01	-0.011	8.24e − 01
	CD11b (ITGAM)	0.439	^∗∗∗^	0.429	^∗∗∗^
	CCR7	0.416	^∗∗∗^	0.39	^∗∗∗^

Natural killer cell	KIR2DL1	0.144	^∗^	0.124	1.61e − 02
	KIR2DL3	0.118	1.64e − 02	0.079	1.27e − 01
	KIR2DL4	-0.043	3.79e − 01	-0.079	1.25e − 01
	KIR3DL1	0.121	1.36e − 02	0.091	7.85e − 02
	KIR3DL2	0.163	^∗∗^	0.121	1.83e − 02
	KIR3DL3	-0.11	2.48e − 02	-0.12	1.98e − 02
	KIR2DS4	0.04	4.20e − 01	0.01	8.41e − 01

Dendritic cell	HLA-DPB1	0.315	^∗∗∗^	0.274	^∗∗∗^
	HLA-DQB1	0.171	^∗∗^	0.125	1.53e − 02
	HLA-DRA	0.211	^∗∗∗^	0.17	^∗∗^
	HLA-DPA1	0.245	^∗∗∗^	0.203	^∗∗∗^
	BDCA-1 (CD1C)	0.492	^∗∗∗^	0.484	^∗∗∗^
	BDCA-4 (NRP1)	0.599	^∗∗∗^	0.591	^∗∗∗^
	CD11c (ITGAX)	0.396	^∗∗∗^	0.368	^∗∗∗^

Th1	T-bet (TBX21)	0.263	^∗∗∗^	0.236	^∗∗∗^
	STAT4	0.322	^∗∗∗^	0.301	^∗∗∗^
	STAT1	0.002	9.64e − 01	-0.013	7.97e − 01
	IFN-*γ* (IFNG)	-0.034	4.89e − 01	-0.06	2.44e − 01
	TNF-*α* (TNF)	0.071	1.46e − 01	0.384	^∗∗∗^

Th2	GATA3	0.397	^∗∗∗^	0.034	5.10e − 01
	STAT6	0.141	^∗^	0.141	^∗^
	STAT5A	0.381	^∗∗∗^	0.369	^∗∗∗^
	IL13	0.16	^∗^	0.172	^∗∗^

Tfh	BCL6	0.497	^∗∗∗^	0.48	^∗∗∗^
	IL21	0.082	9.68e − 02	0.065	2.06e − 01

Th17	STAT3	0.348	^∗∗∗^	0.342	^∗∗∗^
	IL17A	-0.283	^∗∗∗^	-0.297	^∗∗∗^

Treg	FOXP3	0.237	^∗∗∗^	0.202	^∗∗∗^
	CCR8	0.358	^∗∗∗^	0.346	^∗∗∗^
	STAT5B	0.53	^∗∗∗^	0.522	^∗∗∗^
	TGF*β* (TGFB1)	0.6	^∗∗∗^	0.593	^∗∗∗^

T cell exhaustion	PD-1 (PDCD1)	0.171	^∗∗^	0.132	1.02e − 02
	CTLA4	0.111	2.31e − 02	0.074	1.49e − 01
	LAG3	0.143	^∗^	0.109	3.30e − 02
	TIM-3 (HAVCR2)	0.387	^∗∗∗^	0.365	^∗∗∗^
	GZMB	0.068	1.70e − 01	0.017	7.47e − 01

STAD: stomach adenocarcinoma; TAM: tumor-associated macrophage; Th: T helper cell; Tfh: follicular helper T cell; Treg: regulatory T cell. Cor: *R*-value of Spearman's correlation; None: correlation without adjusted; Purity: correlation adjusted by purity. ^∗^*P* < 0.01; ^∗∗^*P* < 0.001; ^∗∗∗^*P* < 0.0001.

**Table 3:** Correlation analysis between PDLIM3 and related gene markers of macrophage, dendritic cell, and Treg in GEPIA.

Description	Gene markers	STAD			
		Tumor		Normal	
		*R*	*P*	*R*	*P*
TAM	CCL2	0.58	^∗∗∗^	0.55	^∗∗^
	CD68	0.22	^∗∗∗^	−0.5	^∗^
	IL10	0.4	^∗∗∗^	0.012	0.95

M2 macrophage	CD163	0.43	^∗∗∗^	0.66	^∗∗∗^
	VSIG4	0.5	^∗∗∗^	0.46	^∗^
	MS4A4A	0.5	^∗∗∗^	0.57	^∗∗^

Dendritic cell	HLA-DPB1	0.29	^∗∗∗^	-0.25	0.13
	HLA-DQB1	0.14	^∗^	-0.48	^∗^
	HLA-DRA	0.2	^∗∗∗^	-0.38	0.022
	HLA-DPA1	0.24	^∗∗∗^	-0.31	0.07
	BDCA-1 (CD1C)	0.49	^∗∗∗^	0.034	0.84
	BDCA-4 (NRP1)	0.58	^∗∗∗^	0.71	^∗∗∗^
	CD11c (ITGAX)	0.38	^∗∗∗^	0.03	0.86

Treg	FOXP3	0.24	^∗∗∗^	-0.59	^∗∗^
	CCR8	0.37	^∗∗∗^	-0.32	0.056
	STAT5B	0.53	^∗∗∗^	0.87	^∗∗∗^
	TGF*β* (TGFB1)	0.59	^∗∗∗^	0.51	^∗∗∗^

Tumor: correlation analysis in tumor tissue; Normal: correlation analysis in normal tissue.

## Data Availability

The datasets investigated in this study are available from the corresponding authors on reasonable request.

## Supplementary Materials

Supplementary Materials Details of IHC, mRNA expression in the Oncomine database, prognostic relations in Prognoscan database are provided.

## References

[1] Frei E. R. (1982). Clinical cancer research: an embattled species. *Cancer*.

[2] Wu H., Wang W., Tong S., Wu C. (2015). Nucleostemin regulates proliferation and migration of gastric cancer and correlates with its malignancy. *International Journal Clinical Experimental Medine*.

[3] Velin D., Michetti P. (2006). Immunology of <i>Helicobacter pylori</i> infection. *Digestion*.

[4] Ito T., Kobayashi D., Uchida K. (2008). _Helicobacter pylori_ invades the gastric mucosa and translocates to the gastric lymph nodes. *Laboratory Investigation*.

[5] Wen S., Felley C. P., Bouzourene H., Reimers M., Michetti P., Pan-Hammarström Q. (2004). Inflammatory gene profiles in gastric mucosa during Helicobacter pyloriInfection in humans. *Journal of Immunology*.

[6] Overman M. J., McDermott R., Leach J. L. (2017). Nivolumab in patients with metastatic DNA mismatch repair-deficient or microsatellite instability-high colorectal cancer (CheckMate 142): an open- label, multicentre, phase 2 study. *Lancet Oncology*.

[7] Le D. T., Uram J. N., Wang H. (2015). PD-1 blockade in tumors with mismatch-repair deficiency. *New England Journal Medicine*.

[8] Muro K., Chung H. C., Shankaran V. (2016). Pembrolizumab for patients with PD-L1-positive advanced gastric cancer (KEYNOTE-012): a multicentre, open-label, phase 1b trial. *Lancet Oncology*.

[9] Zhang H., Liu H., Shen Z. (2018). Tumor-infiltrating neutrophils is prognostic and predictive for postoperative adjuvant chemotherapy benefit in patients with gastric cancer. *Annals of Surgery*.

[10] Xia H., Winokur S. T., Kuo W. L., Altherr M. R., Bredt D. S. (1997). Actinin-associated LIM protein: identification of a domain interaction between PDZ and spectrin-like repeat motifs. *Journal of Cell Biology*.

[11] Ohsawa N., Koebis M., Suo S., Nishino I., Ishiura S. (2011). Alternative splicing of _PDLIM3/ALP_ , for *α*-actinin-associated LIM protein 3, is aberrant in persons with myotonic dystrophy. *Biochemical Biophysical Research Communications*.

[12] Bouju S., Piétu G., le Cunff M. (1999). Exclusion of muscle specific actinin-associated LIM protein (ALP) gene from 4q35 facioscapulohumeral muscular dystrophy (FSHD) candidate genes. *Neuromuscular Disorders*.

[13] Yin H., Zhao J., He H. (2020). Gga-miR-3525 targets PDLIM3 through the MAPK signaling pathway to regulate the proliferation and differentiation of skeletal muscle satellite cells. *International Journal of Molecular Sciences*.

[14] Wilde A. A. M., Behr E. R. (2013). Genetic testing for inherited cardiac disease. *Nature Reviews Cardiology*.

[15] Wang D., Fang J., Lv J. (2019). Novel polymorphisms in PDLIM3 and PDLIM5 gene encoding Z-line proteins increase risk of idiopathic dilated cardiomyopathy. *Journal of Cellular Molecular Medicine*.

[16] Lak N. S. M., Voormanns T. L., Zappeij-Kannegieter L. (2021). Improving risk stratification for pediatric patients with rhabdomyosarcoma by molecular detection of disseminated disease. *Clinical Cancer Research*.

[17] Lee D. Y., Kang Y., Im N. R. (2021). Actin-associated gene expression is associated with early regional metastasis of tongue cancer. *Laryngoscope*.

[18] Shou Y., Robinson D. M., Amakye D. D. (2015). A five-gene hedgehog signature developed as a patient preselection tool for hedgehog inhibitor therapy in medulloblastoma. *Clinical Cancer Research*.

[19] Feng Y., Jiang Y., Wen T., Meng F., Shu X. (2020). Identifying potential prognostic markers for muscle-invasive bladder urothelial carcinoma by weighted gene co-expression network analysis. *Pathology & Oncology Research*.

[20] Chae Y. K., Chang S., Ko T. (2018). Epithelial-mesenchymal transition (EMT) signature is inversely associated with T-cell infiltration in non-small cell lung cancer (NSCLC). *Scientific Reports*.

[21] Li Y., Rong G., Kang H. (2017). Taxotere-induced elevated expression of IL8 in carcinoma-associated fibroblasts of breast invasive ductal cancer. *Oncology Letters*.

[22] Duggal N. A., Niemiro G., Harridge S. D. R., Simpson R. J., Lord J. M. (2019). Can physical activity ameliorate immunosenescence and thereby reduce age- related multi-morbidity?. *Nature Reviews Immunology*.

[23] Higashino N., Koma Y. I., Hosono M. (2019). Fibroblast activation protein-positive fibroblasts promote tumor progression through secretion of CCL2 and interleukin-6 in esophageal squamous cell carcinoma. *Laboratory Investigation*.

[24] Tanaka T., Yamamoto Y., Muromoto R. (2011). PDLIM2 inhibits T helper 17 cell development and granulomatous inflammation through degradation of STAT3. *Science Signaling*.

[25] Rhodes D. R., Kalyana-Sundaram S., Mahavisno V. (2007). Oncomine 3.0: genes, pathways, and networks in a collection of 18,000 cancer gene expression profiles. *Neoplasia*.

[26] Thul P. J., Lindskog C. (2018). The human protein atlas: a spatial map of the human proteome. *Protein Science*.

[27] Lánczky A., Nagy Á., Bottai G. (2016). miRpower: a web-tool to validate survival-associated miRNAs utilizing expression data from 2178 breast cancer patients. *Breast Cancer Research and Treatment*.

[28] Mizuno H., Kitada K., Nakai K., Sarai A. (2009). PrognoScan: a new database for meta-analysis of the prognostic value of genes. *BMC Medical Genomics*.

[29] Li T., Fan J., Wang B. (2017). TIMER: a web server for comprehensive analysis of tumor-infiltrating immune cells. *Cancer Research*.

[30] Tang Z., Li C., Kang B., Gao G., Li C., Zhang Z. (2017). GEPIA: a web server for cancer and normal gene expression profiling and interactive analyses. *Nucleic Acids Research*.

[31] Li B., Severson E., Pignon J. C. (2016). Comprehensive analyses of tumor immunity: implications for cancer immunotherapy. *Genome Biology*.

[32] Ohtani H. (2007). Focus on TILs: prognostic significance of tumor infiltrating lymphocytes in human colorectal cancer. *Cancer Immunotherapy*.

[33] Azimi F., Scolyer R. A., Rumcheva P. (2012). Tumor-infiltrating lymphocyte grade is an independent predictor of sentinel lymph node status and survival in patients with cutaneous melanoma. *Journal of Clinical Oncology*.

[34] Zhang N., Cao M., Duan Y., Bai H., Li X., Wang Y. (2020). Prognostic role of tumor-infiltrating lymphocytes in gastric cancer: a meta-analysis and experimental validation. *Archives of Medicine Science*.

[35] Mantovani A., Marchesi F., Malesci A., Laghi L., Allavena P. (2017). Tumour-associated macrophages as treatment targets in oncology. *Nature Reviews Clinical Oncology*.

[36] Lewis C. E., Pollard J. W. (2006). Distinct role of macrophages in different tumor microenvironments. *Cancer Research*.

[37] Bercovici N., Guérin M. V., Trautmann A., Donnadieu E. (2019). The remarkable plasticity of macrophages: a chance to fight cancer. *Frontiers in Immunology*.

[38] Dagouassat M., Suffee N., Hlawaty H. (2010). Monocyte chemoattractant protein-1 (MCP-1)/CCL2 secreted by hepatic myofibroblasts promotes migration and invasion of human hepatoma cells. *International Journal of Cancer*.

[39] Tang C. H., Tsai C. C. (2012). CCL2 increases MMP-9 expression and cell motility in human chondrosarcoma cells via the Ras/Raf/MEK/ERK/NF-*κ*B signaling pathway. *Biochemical Pharmacology*.

[40] Liao Y., Guo S., Chen Y. (2014). VSIG4 expression on macrophages facilitates lung cancer development. *Laboratory Investigation*.

[41] Mattiola I., Tomay F., de Pizzol M. (2019). The macrophage tetraspan MS4A4A enhances dectin-1-dependent NK cell-mediated resistance to metastasis. *Nature Immunology*.

[42] Ohue Y., Nishikawa H. (2019). Regulatory T (Treg) cells in cancer: can Treg cells be a new therapeutic target?. *Cancer Science*.

[43] Zhang Y., Lazarus J., Steele N. G. (2020). Regulatory T-cell depletion alters the tumor microenvironment and accelerates pancreatic carcinogenesis. *Cancer Discovery*.

[44] Garris C. S., Arlauckas S. P., Kohler R. H. (2018). Successful anti-PD-1 cancer immunotherapy requires T cell-dendritic cell crosstalk involving the cytokines IFN-*γ* and IL-12. *Immunity*.

[45] Nizzoli G., Krietsch J., Weick A. (2013). Human CD1c+ dendritic cells secrete high levels of IL-12 and potently prime cytotoxic T-cell responses. *Blood*.

[46] Berdiel-Acer M., Berenguer A., Sanz-Pamplona R. (2014). A 5-gene classifier from the carcinoma-associated fibroblast transcriptomic profile and clinical outcome in colorectal cancer. *Oncotarget*.

[47] Porta C., Paglino C., Mosca A. (2014). Targeting PI3K/Akt/mTOR signaling in cancer. *Frontiers in Oncology*.

[48] Jafari M., Ghadami E., Dadkhah T., Niaki H. A. (2019). PI3k/AKT signaling pathway: erythropoiesis and beyond. *Journal of Cellular Physiology*.

[49] Le Rhun E., Bertrand N., Dumont A. (2017). Identification of single nucleotide polymorphisms of the PI3K-AKT-mTOR pathway as a risk factor of central nervous system metastasis in metastatic breast cancer. *European Journal of Cancer*.

[50] Cuarental L., Sucunza-Sáenz D., Valiño-Rivas L. (2019). MAP3K quinasas y daño renal. *Nefrología suplemento extraordinario*.

[51] Igea A., Nebreda A. R. (2015). The stress kinase p38*α* as a target for cancer therapy. *Cancer Research*.

[52] Wu X., Zhang W., Font-Burgada J. (2014). Ubiquitin-conjugating enzyme Ubc13 controls breast cancer metastasis through a TAK1-p38 MAP kinase cascade. *PNAS Nexus*.

[53] Barrantes I. B., Nebreda A. R. (2012). Roles of p38 MAPKs in invasion and metastasis. *Biochemical Society Transactions*.

[54] Zhou S. L., Zhou Z. J., Hu Z. Q. (2016). Tumor-associated neutrophils recruit macrophages and T-regulatory cells to promote progression of hepatocellular carcinoma and resistance to sorafenib. *Gastroenterology*.

[55] Gu J., Qian H., Shen L. (2012). Gastric cancer exosomes trigger differentiation of umbilical cord derived mesenchymal stem cells to carcinoma-associated fibroblasts through TGF-*β*/Smad pathway. *PLoS One*.

