# Supplementary material for: Comprehensive Analysis of PDLIM3 Expression Profile, Prognostic Value, and Correlations with Immune Infiltrates in Gastric Cancer
Source: J Immunol Res. 2022 May 12;2022:2039447. doi: 10.1155/2022/2039447 (PMC9135576; doi:10.1155/2022/2039447)
Supplement: Supplementary Materials — Details of IHC, mRNA expression in the Oncomine database, prognostic relations in Prognoscan database are provided. [file 2039447.f1.doc]

**Supplementary Materials**

**Comprehensive Analysis of PDLIM3 Expression Profile, Prognostic Value and Correlations with Immune Infiltrates in Gastric Cancer**

Table S1. Clinical information of 15 gastric cancer samples for immunohistochemistry.

| Sample no. | Gender | Age (years) | Pathological type |
| --- | --- | --- | --- |
| S1 | F | 44 | Adenocarcinoma |
| S2 | M | 66 | Adenocarcinoma |
| S3 | M | 74 | Adenocarcinoma |
| S4 | F | 64 | Adenocarcinoma |
| S5 | F | 63 | Adenocarcinoma |
| S6 | M | 68 | Adenocarcinoma |
| S7 | M | 74 | Adenocarcinoma |
| S8 | F | 47 | Adenocarcinoma |
| S9 | M | 64 | Squamous carcinoma |
| S10 | M | 63 | Squamous carcinoma |
| S11 | M | 74 | Squamous carcinoma |
| S12 | M | 65 | Squamous carcinoma |
| S13 | M | 55 | Squamous carcinoma |
| S14 | M | 75 | Squamous carcinoma |
| S15 | M | 56 | Squamous carcinoma |

Table S2. PDLIM3 expression in cancers versus normal tissues in Oncomine database.

| Cancer | Cancer | *P*-value | Fold | Rank | t‑Test | Sample | Reference |
| --- | --- | --- | --- | --- | --- | --- | --- |
|  | type |  | change | (%) |  |  | (PMID) |
| Bladder | Infiltrating Bladder Urothelial Carcinoma | 8.36E-05 | 59.254 | 1% | 7.486 | 6 | Sanchez-Carbayo Bladder |
|  | Infiltrating Bladder Urothelial Carcinoma | 7.33E-25 | -9.236 | 1% | -12.81 | 48 | Sanchez-Carbayo Bladder |
|  | Superficial Bladder Cancer | 2.46E-05 | -8.837 | 2% | -10.79 | 15 | Sanchez-Carbayo Bladder |
|  | Superficial Bladder Cancer | 8.89E-16 | -5.507 | 1% | -9.854 | 126 | [31515488](https://www.ncbi.nlm.nih.gov/pubmed/31515488) |
|  | Infiltrating Bladder Urothelial Carcinoma | 6.02E-07 | -3.075 | 5% | -5.1 | 62 | [31515488](https://www.ncbi.nlm.nih.gov/pubmed/31515488) |
|  | Superficial Bladder Cancer | 6.60E-06 | -2.846 | 8% | -5.152 | 28 | Dyrskjot Bladder |
| Brain and  CNS | Glioblastoma | 2.37E-14 | 2.885 | 3% | 9.577 | 81 | Sun Brain |
| Breast | Lobular Breast Carcinoma | 6.67E-09 | -2.787 | 1% | -9.17 | 19 | Zhao Breast |
|  | Invasive Ductal Breast Carcinoma | 8.64E-10 | -2.674 | 1% | 0.01 | 34 | Zhao Breast |
|  | Ductal Breast Carcinoma in Situ | 3.07E-05 | -3.979 | 2% | -4.99 | 9 | Ma Breast |
|  | Ductal Breast Carcinoma | 2.55E-08 | -8.095 | 3% | -11.19 | 40 | Richardson Breast |
|  | Invasive Ductal and Lobular Carcinoma | 8.47E-05 | -2.289 | 4% | -6.083 | 3 | TCGA |
|  | Invasive Lobular Breast Carcinoma | 4.08E-11 | -3.26 | 3% | -7.428 | 36 | TCGA |
|  | Invasive Breast Carcinoma | 2.64E-12 | -2.773 | 9% | -7.718 | 76 | TCGA |
|  | Medullary Breast Carcinoma | 4.27E-11 | -2.854 | 6% | -8.216 | 32 | [26214276](https://www.ncbi.nlm.nih.gov/pubmed/26214276) |
|  | Mucinous Breast Carcinoma | 1.40E-14 | -2.866 | 7% | -9.24 | 46 | [31515488](https://www.ncbi.nlm.nih.gov/pubmed/31515488) |
|  | Invasive Lobular Breast Carcinoma | 2.21E-22 | -2.236 | 8% | -10.54 | 148 | [31515488](https://www.ncbi.nlm.nih.gov/pubmed/31515488) |
| Colorectal | Colon Adenoma | 3.17E-10 | -3.401 | 1% | -17.55 | 5 | Skrzypczak Colorectal |
|  | Colon Adenoma | 5.65E-08 | -3.355 | 4% | -6.709 | 25 | Sabates-Bellver Colorectal |
| Esophageal | Esophageal Adenocarcinoma | 1.30E-12 | 2.38 | 4% | 7.966 | 75 | [31515488](https://www.ncbi.nlm.nih.gov/pubmed/31515488) |
| Gastric | Diffuse Gastric Adenocarcinoma | 3.22E-05 | 2.269 | 1% | 4.159 | 47 | [12477932](https://www.ncbi.nlm.nih.gov/pubmed/12477932) |
| Head and neck | Tongue Squamous Cell Carcinoma | 5.48E-05 | -5.908 | 7% | -4.233 | 31 | Estilo Head-Neck |
| Kidney | Hereditary Clear Cell Renal Cell Carcinoma | 2.57E-08 | 3.067 | 6% | 7.002 | 32 | Beroukhim Kidney |
|  | Clear Cell Sarcoma of the Kidney | 9.46E-05 | -3.087 | 4% | -4.942 | 14 | Cutcliffe Kidney |
|  | Renal Oncocytoma | 7.63E-09 | -3.163 | 7% | -12.02 | 12 | [12477932](https://www.ncbi.nlm.nih.gov/pubmed/12477932) |
|  | Clear Cell Renal Cell Carcinoma | 2.26E-10 | -2.307 | 8% | -9.408 | 23 | [12477932](https://www.ncbi.nlm.nih.gov/pubmed/12477932) |
|  | Renal Pelvis Urothelial Carcinoma | 3.26E-06 | -10.466 | 8% | -10.47 | 8 | [12477932](https://www.ncbi.nlm.nih.gov/pubmed/12477932) |
| Liver | Cirrhosis | 2.05E-17 | 2.48 | 2% | 12.297 | 58 | Mas Liver |
|  | Hepatocellular Carcinoma | 1.98E-10 | 2.204 | 2% | 7.628 | 38 | Mas Liver |
| Lung | Lung Adenocarcinoma | 1.26E-06 | -2.285 | 2% | -6.129 | 38 | Garber Lung |
|  | Squamous Cell Lung Carcinoma | 7.20E-05 | -5.632 | 4% | -4.303 | 21 | Bhattacharjee Lung |
|  | Lung Carcinoid Tumor | 2.10E-07 | -11.193 | 7% | -6.679 | 20 | Bhattacharjee Lung |
|  | Squamous Cell Lung Carcinoma | 9.08E-08 | -6.687 | 4% | -7.393 | 26 | Talbot Lung |
|  | Lung Adenocarcinoma | 5.14E-18 | -2.495 | 4% | -10.28 | 58 | [26214276](https://www.ncbi.nlm.nih.gov/pubmed/26214276) |
|  | Lung Adenocarcinoma | 1.82E-11 | -2.909 | 7% | -7.914 | 45 | [12477932](https://www.ncbi.nlm.nih.gov/pubmed/12477932) |
|  | Large Cell Lung Carcinoma | 1.10E-07 | -3.629 | 9% | -7.255 | 19 | [12477932](https://www.ncbi.nlm.nih.gov/pubmed/12477932) |
| Lymphoma | Centroblastic Lymphoma | 3.94E-16 | 12.041 | 2% | 12.601 | 28 | Basso Lymphoma |
|  | Diffuse Large B-Cell Lymphoma | 6.30E-05 | 3.433 | 8% | 4.146 | 32 | Basso Lymphoma |
|  | Follicular Lymphoma | 8.89E-22 | 2.803 | 2% | 15.937 | 38 | Compagno Lymphoma |
|  | Germinal Center B-Cell-Like Diffuse Large B-Cell Lymphoma | 1.10E-06 | 2.882 | 3% | 10.24 | 9 | Compagno Lymphoma |
|  | Diffuse Large B-Cell Lymphoma | 1.69E-13 | 4.007 | 6% | 10.009 | 44 | Compagno Lymphoma |
|  | Activated B-Cell-Like Diffuse Large B-Cell Lymphoma | 3.16E-07 | 2.532 | 9% | 7.518 | 17 | Compagno Lymphoma |
|  | Unspecified Peripheral T-Cell Lymphoma | 2.21E-10 | 3.278 | 6% | 8.921 | 28 | Piccaluga Lymphoma |
|  | Diffuse Large B-Cell Lymphoma | 3.62E-05 | -10.497 | 1% | -6.465 | 6 | Storz Lymphoma |
| Ovarian | Ovarian Serous Adenocarcinoma | 2.92E-15 | -41.43 | 2% | -12.51 | 43 | [31515488](https://www.ncbi.nlm.nih.gov/pubmed/31515488) |
|  | Ovarian Serous Cystadenocarcinoma | 1.15E-05 | -3.945 | 4% | -9.002 | 586 | TCGA |
| Pancreatic | Pancreatic Ductal Adenocarcinoma | 2.25E-10 | 2.712 | 4% | 7.626 | 39 | Badea pancreatic |
| Prostate | Prostate Carcinoma | 4.18E-05 | -2.391 | 3% | -4.578 | 65 | Yu Prostate |
| Sarcoma | Leiomyosarcoma | 3.32E-05 | 8.261 | 3% | 5.082 | 6 | Detwille Sarcoma |
|  | Leiomyosarcoma | 6.95E-08 | 10.169 | 6% | 8.27 | 26 | Barretina Sarcoma |
|  | Clear Cell Sarcoma of the Kidney | 9.46E-05 | -3.087 | 4% | -4.942 | 14 | Cutcliffe Sarcoma |

Table S3. Relation between PDLIM3 expression and patient prognosis of different cancers in Prognoscan database.

| Cancer type | Data set | Endpoint | N | HR [95% CI-low CI-up] | Cox *P*-Value |
| --- | --- | --- | --- | --- | --- |
| Bladder cancer | GSE5287 | OS | 30 | 1.04 [0.70 - 1.52] | 0.856703 |
|  | GSE5287 |  | 30 | 0.62 [0.25 - 1.52] | 0.299602 |
|  | GSE13507 |  | 165 | 0.99 [0.83 - 1.18] | 0.905409 |
|  | GSE13507 | DFS | 165 | 1.11 [0.90 - 1.37] | 0.314573 |
| Blood cancer | GSE12417-GPL96 | OS | 163 | 1.77 [0.40 - 7.76] | 0.447338 |
|  | GSE12417-GPL96 |  | 163 | 0.85 [0.23 - 3.13] | 0.807138 |
|  | GSE12417-GPL97 |  | 163 | 1.25 [0.24 - 6.49] | 0.792733 |
|  | GSE12417-GPL570 |  | 79 | 1.17 [0.28 - 4.85] | 0.830363 |
|  | GSE12417-GPL570 |  | 79 | 15.76 [1.44 - 172.14] | 0.0237887 |
|  | GSE12417-GPL570 |  | 79 | 0.09 [0.02 - 0.43] | 0.00269973 |
|  | GSE5122 |  | 58 | 1.14 [0.80 - 1.62] | 0.462666 |
|  | GSE5122 |  | 58 | 0.86 [0.64 - 1.17] | 0.342617 |
|  | GSE8970 |  | 34 | 0.54 [0.29 - 0.99] | 0.0455636 |
|  | GSE8970 |  | 34 | 0.89 [0.61 - 1.30] | 0.540029 |
|  | GSE4475 |  | 158 | 0.47 [0.27 - 0.81] | 0.00681718 |
|  | GSE4475 |  | 158 | 0.31 [0.10 - 0.95] | 0.0406125 |
|  | E-TABM-346 | EFS | 53 | 0.86 [0.51 - 1.46] | 0.571037 |
|  | E-TABM-346 | OS | 53 | 0.87 [0.60 - 1.26] | 0.466335 |
|  | E-TABM-346 | EFS | 53 | 0.90 [0.64 - 1.28] | 0.570748 |
|  | E-TABM-346 | OS | 53 | 0.87 [0.51 - 1.50] | 0.619382 |
|  | GSE16131-GPL96 |  | 180 | 0.79 [0.57 - 1.09] | 0.147367 |
|  | GSE16131-GPL96 |  | 180 | 0.89 [0.73 - 1.08] | 0.229356 |
|  | GSE16131-GPL97 |  | 180 | 1.04 [0.89 - 1.20] | 0.63377 |
|  | GSE2658 | DSS | 559 | 0.99 [0.82 - 1.18] | 0.880366 |
|  | GSE2658 |  | 559 | 0.90 [0.76 - 1.07] | 0.239735 |
|  | GSE2658 |  | 559 | 0.96 [0.84 - 1.10] | 0.561034 |
|  | GSE4271-GPL96 | OS | 77 | 1.66 [1.19 - 2.34] | 0.00323873 |
|  | GSE4271-GPL96 |  | 77 | 1.38 [0.93 - 2.04] | 0.106182 |
|  | GSE4271-GPL97 |  | 77 | 1.09 [0.83 - 1.42] | 0.537396 |
|  | GSE7696 |  | 70 | 0.94 [0.52 - 1.70] | 0.840657 |
|  | GSE7696 |  | 70 | 1.69 [0.60 - 4.74] | 0.319819 |
|  | GSE7696 |  | 70 | 1.13 [0.85 - 1.49] | 0.406637 |
|  | MGH-glioma |  | 50 | 1.86 [1.01 - 3.43] | 0.04647 |
|  | GSE4412-GPL96 |  | 74 | 1.76 [1.23 - 2.52] | 0.00184419 |
|  | GSE4412-GPL96 |  | 74 | 1.42 [0.94 - 2.13] | 0.093524 |
|  | GSE4412-GPL97 |  | 74 | 1.28 [0.95 - 1.72] | 0.104085 |
|  | GSE16581 |  | 67 | 0.01 [0.00 - 3.13] | 0.114971 |
|  | GSE16581 |  | 67 | 2.55 [0.00 - 4879.14] | 0.808022 |
|  | GSE16581 |  | 67 | 0.30 [0.03 - 2.96] | 0.30095 |
| Breast cancer | GSE19615 | DMFS | 115 | 1.91 [0.46 - 7.96] | 0.373796 |
|  | GSE19615 |  | 115 | 1.60 [0.70 - 3.69] | 0.265747 |
|  | GSE19615 |  | 115 | 1.47 [0.64 - 3.38] | 0.367413 |
|  | GSE3143 | OS | 158 | 1.05 [0.70 - 1.57] | 0.815017 |
|  | GSE7849 | DFS | 76 | 1.05 [0.53 - 2.08] | 0.896123 |
|  | GSE12276 | RFS | 204 | 1.29 [1.09 - 1.53] | 0.00316731 |
|  | GSE12276 |  | 204 | 1.25 [1.08 - 1.45] | 0.00348059 |
|  | GSE12276 |  | 204 | 1.15 [0.97 - 1.36] | 0.111086 |
|  | GSE6532-GPL570 | DMFS | 87 | 0.78 [0.57 - 1.08] | 0.139291 |
|  | GSE6532-GPL570 | RFS | 87 | 0.80 [0.47 - 1.36] | 0.40082 |
|  | GSE6532-GPL570 | DMFS | 87 | 0.80 [0.47 - 1.36] | 0.40082 |
|  | GSE6532-GPL570 | RFS | 87 | 0.82 [0.61 - 1.12] | 0.218904 |
|  | GSE6532-GPL570 |  | 87 | 0.78 [0.57 - 1.08] | 0.139291 |
|  | GSE6532-GPL570 | DMFS | 87 | 0.82 [0.61 - 1.12] | 0.218904 |
|  | GSE9195 | RFS | 77 | 1.31 [0.69 - 2.48] | 0.407766 |
|  | GSE9195 | DMFS | 77 | 1.20 [0.63 - 2.29] | 0.576382 |
|  | GSE9195 | RFS | 77 | 1.10 [0.63 - 1.95] | 0.732923 |
|  | GSE9195 | DMFS | 77 | 1.59 [0.81 - 3.13] | 0.18062 |
|  | GSE9195 | RFS | 77 | 1.08 [0.61 - 1.91] | 0.80328 |
|  | GSE9195 | DMFS | 77 | 1.49 [0.81 - 2.76] | 0.202732 |
|  | GSE12093 |  | 136 | 1.15 [0.61 - 2.14] | 0.669105 |
|  | GSE12093 |  | 136 | 0.91 [0.53 - 1.59] | 0.746747 |
|  | GSE11121 |  | 200 | 1.03 [0.63 - 1.67] | 0.906751 |
|  | GSE11121 |  | 200 | 1.18 [0.72 - 1.93] | 0.509343 |
|  | GSE1378 | RFS | 60 | 1.07 [0.89 - 1.29] | 0.465992 |
|  | GSE1379 |  | 60 | 1.08 [0.77 - 1.51] | 0.641282 |
|  | GSE2034 | DMFS | 286 | 0.96 [0.73 - 1.26] | 0.757147 |
|  | GSE2034 |  | 286 | 0.86 [0.65 - 1.16] | 0.326312 |
|  | GSE1456-GPL96 | OS | 159 | 0.77 [0.49 - 1.21] | 0.261201 |
|  | GSE1456-GPL96 | RFS | 159 | 0.90 [0.56 - 1.47] | 0.684391 |
|  | GSE1456-GPL96 | DSS | 159 | 0.97 [0.54 - 1.75] | 0.920793 |
|  | GSE1456-GPL96 | RFS | 159 | 0.75 [0.48 - 1.19] | 0.223477 |
|  | GSE1456-GPL96 | OS | 159 | 0.73 [0.45 - 1.18] | 0.193643 |
|  | GSE1456-GPL96 | DSS | 159 | 0.78 [0.46 - 1.33] | 0.358055 |
|  | GSE1456-GPL97 | OS | 159 | 0.87 [0.62 - 1.22] | 0.426036 |
|  | GSE1456-GPL97 | RFS | 159 | 1.01 [0.71 - 1.43] | 0.967209 |
|  | GSE1456-GPL97 | DSS | 159 | 0.98 [0.65 - 1.48] | 0.918999 |
|  | GSE7378 | DFS | 54 | 1.42 [0.76 - 2.65] | 0.269809 |
|  | GSE7378 |  | 54 | 1.07 [0.48 - 2.35] | 0.872306 |
|  | E-TABM-158 | RFS | 117 | 1.02 [0.77 - 1.34] | 0.914157 |
|  | E-TABM-158 | DFS | 117 | 1.10 [0.80 - 1.52] | 0.545575 |
|  | E-TABM-158 | OS | 117 | 1.02 [0.77 - 1.34] | 0.914157 |
|  | E-TABM-158 | DMFS | 117 | 0.98 [0.69 - 1.39] | 0.902749 |
|  | E-TABM-158 | RFS | 117 | 1.12 [0.81 - 1.55] | 0.478519 |
|  | E-TABM-158 | DSS | 117 | 1.11 [0.76 - 1.63] | 0.594958 |
|  | E-TABM-158 | DMFS | 117 | 1.05 [0.68 - 1.61] | 0.837078 |
|  | E-TABM-158 | OS | 117 | 1.12 [0.81 - 1.55] | 0.478519 |
|  | GSE3494-GPL96 | DSS | 236 | 1.08 [0.70 - 1.65] | 0.732544 |
|  | GSE3494-GPL96 |  | 236 | 0.87 [0.55 - 1.38] | 0.554786 |
|  | GSE3494-GPL97 |  | 236 | 1.00 [0.76 - 1.30] | 0.976831 |
|  | GSE4922-GPL96 | DFS | 249 | 0.81 [0.56 - 1.16] | 0.248717 |
|  | GSE4922-GPL96 |  | 249 | 0.87 [0.61 - 1.22] | 0.414744 |
|  | GSE4922-GPL97 |  | 249 | 0.86 [0.70 - 1.06] | 0.161326 |
|  | GSE2990 | RFS | 125 | 0.74 [0.44 - 1.24] | 0.252032 |
|  | GSE2990 | DMFS | 54 | 0.81 [0.37 - 1.80] | 0.60758 |
|  | GSE2990 | RFS | 62 | 0.65 [0.37 - 1.15] | 0.137438 |
|  | GSE2990 | DMFS | 125 | 1.16 [0.72 - 1.86] | 0.543259 |
|  | GSE2990 | RFS | 62 | 0.77 [0.40 - 1.49] | 0.434902 |
|  | GSE2990 |  | 125 | 0.90 [0.63 - 1.30] | 0.587572 |
|  | GSE2990 | DMFS | 54 | 0.64 [0.32 - 1.27] | 0.202239 |
|  | GSE2990 |  | 125 | 0.85 [0.43 - 1.67] | 0.639406 |
|  | GSE7390 | RFS | 198 | 1.22 [0.99 - 1.49] | 0.0585636 |
|  | GSE7390 | DMFS | 198 | 1.22 [0.97 - 1.54] | 0.0957401 |
|  | GSE7390 | OS | 198 | 1.16 [0.91 - 1.48] | 0.223421 |
|  | GSE7390 | DMFS | 198 | 1.37 [1.07 - 1.75] | 0.0116095 |
|  | GSE7390 | OS | 198 | 1.36 [1.05 - 1.76] | 0.0193056 |
|  | GSE7390 | RFS | 198 | 1.12 [0.93 - 1.35] | 0.245955 |
| Colorectal cancer | GSE12945 | OS | 62 | 0.18 [0.00 - 24.61] | 0.495948 |
|  | GSE12945 | DFS | 51 | 0.68 [0.09 - 5.38] | 0.718741 |
|  | GSE12945 | OS | 62 | 0.94 [0.33 - 2.69] | 0.913058 |
|  | GSE12945 | DFS | 51 | 33.11 [0.18 - 5998.40] | 0.187076 |
|  | GSE17536 | OS | 177 | 1.30 [0.99 - 1.70] | 0.0551692 |
|  | GSE17536 | DFS | 145 | 1.81 [1.25 - 2.62] | 0.00154528 |
|  | GSE17536 | OS | 177 | 1.30 [1.02 - 1.68] | 0.0372193 |
|  | GSE17536 | DSS | 177 | 1.54 [1.17 - 2.02] | 0.00225393 |
|  | GSE17536 | DFS | 145 | 1.66 [1.11 - 2.46] | 0.0130043 |
|  | GSE17536 |  | 145 | 1.86 [1.30 - 2.65] | 0.00060648 |
|  | GSE17536 | DSS | 177 | 1.49 [1.14 - 1.94] | 0.00323107 |
|  | GSE17536 | OS | 177 | 1.32 [1.00 - 1.76] | 0.0526599 |
|  | GSE17536 | DSS | 177 | 1.50 [1.13 - 1.99] | 0.00488249 |
|  | GSE14333 | DFS | 226 | 1.54 [1.19 - 1.99] | 0.00112911 |
|  | GSE14333 |  | 226 | 1.59 [1.25 - 2.04] | 0.000197972 |
|  | GSE14333 |  | 226 | 1.43 [1.16 - 1.74] | 0.000578097 |
|  | GSE17537 |  | 55 | 1.79 [0.43 - 7.44] | 0.425291 |
|  | GSE17537 | OS | 55 | 1.56 [1.03 - 2.37] | 0.0375742 |
|  | GSE17537 | DFS | 55 | 1.86 [0.59 - 5.83] | 0.28598 |
|  | GSE17537 | DSS | 49 | 1.90 [0.37 - 9.64] | 0.440032 |
|  | GSE17537 |  | 49 | 1.42 [0.35 - 5.71] | 0.625543 |
|  | GSE17537 | DFS | 55 | 1.47 [0.91 - 2.37] | 0.117897 |
|  | GSE17537 | DSS | 49 | 1.64 [0.98 - 2.73] | 0.0585809 |
|  | GSE17537 | OS | 55 | 1.67 [0.57 - 4.85] | 0.349466 |
|  | GSE17537 |  | 55 | 1.41 [0.34 - 5.84] | 0.636993 |
| Eye cancer | GSE22138 | DMFS | 63 | 0.90 [0.77 - 1.05] | 0.192266 |
|  | GSE22138 |  | 63 | 0.79 [0.52 - 1.20] | 0.265576 |
|  | GSE22138 |  | 63 | 0.49 [0.17 - 1.37] | 0.173058 |
| Head and neck cancer | GSE2837 | RFS | 28 | 1.53 [0.48 - 4.86] | 0.466285 |
|  | GSE2837 |  | 28 | 0.42 [0.01 - 19.29] | 0.658723 |
|  | GSE2837 |  | 28 | 1.01 [0.24 - 4.29] | 0.991589 |
|  | GSE2837 |  | 28 | 1.39 [0.83 - 2.31] | 0.207197 |
|  | GSE2837 |  | 28 | 0.10 [0.01 - 1.56] | 0.101286 |
| Lung cancer | jacob-00182-CANDF | OS | 82 | 1.41 [0.70 - 2.85] | 0.334885 |
|  | jacob-00182-CANDF |  | 82 | 1.23 [0.78 - 1.94] | 0.37128 |
|  | HARVARD-LC |  | 84 | 1.25 [0.86 - 1.82] | 0.236899 |
|  | jacob-00182-HLM |  | 79 | 1.20 [0.71 - 2.02] | 0.497613 |
|  | jacob-00182-HLM |  | 79 | 1.02 [0.73 - 1.44] | 0.897743 |
|  | jacob-00182-MSK |  | 104 | 0.89 [0.58 - 1.37] | 0.603475 |
|  | jacob-00182-MSK |  | 104 | 0.94 [0.37 - 2.38] | 0.899407 |
|  | GSE13213 |  | 117 | 0.95 [0.72 - 1.24] | 0.693215 |
|  | GSE31210 |  | 204 | 0.87 [0.60 - 1.25] | 0.439672 |
|  | GSE31210 |  | 204 | 1.07 [0.68 - 1.69] | 0.773423 |
|  | GSE31210 | RFS | 204 | 0.82 [0.63 - 1.07] | 0.148996 |
|  | GSE31210 |  | 204 | 0.82 [0.59 - 1.14] | 0.231335 |
|  | GSE31210 | OS | 204 | 1.16 [0.66 - 2.06] | 0.60468 |
|  | GSE31210 | RFS | 204 | 0.85 [0.57 - 1.26] | 0.40833 |
|  | jacob-00182-UM | OS | 178 | 1.19 [0.73 - 1.94] | 0.486375 |
|  | jacob-00182-UM |  | 178 | 1.02 [0.77 - 1.34] | 0.913994 |
|  | GSE3141 |  | 111 | 0.89 [0.67 - 1.20] | 0.449388 |
|  | GSE3141 |  | 111 | 0.93 [0.67 - 1.29] | 0.668182 |
|  | GSE3141 |  | 111 | 1.03 [0.83 - 1.28] | 0.777797 |
|  | GSE14814 |  | 90 | 0.74 [0.30 - 1.80] | 0.503902 |
|  | GSE14814 | DSS | 90 | 0.54 [0.17 - 1.78] | 0.31366 |
|  | GSE14814 | OS | 90 | 1.10 [0.66 - 1.83] | 0.701817 |
|  | GSE14814 | DSS | 90 | 0.99 [0.55 - 1.79] | 0.97717 |
|  | GSE8894 | RFS | 138 | 0.92 [0.73 - 1.15] | 0.45426 |
|  | GSE8894 |  | 138 | 1.03 [0.89 - 1.19] | 0.735748 |
|  | GSE8894 |  | 138 | 0.82 [0.55 - 1.24] | 0.346872 |
|  | GSE4573 | OS | 129 | 1.00 [0.66 - 1.52] | 0.994233 |
|  | GSE4573 |  | 129 | 1.23 [0.82 - 1.83] | 0.312247 |
|  | GSE17710 | RFS | 56 | 1.10 [0.65 - 1.88] | 0.716631 |
|  | GSE17710 | OS | 56 | 0.93 [0.63 - 1.37] | 0.711372 |
|  | GSE17710 | RFS | 56 | 1.12 [0.71 - 1.79] | 0.621529 |
|  | GSE17710 | OS | 56 | 1.07 [0.64 - 1.79] | 0.795726 |
|  | GSE17710 | RFS | 56 | 1.13 [0.83 - 1.53] | 0.450758 |
|  | GSE17710 | OS | 56 | 1.08 [0.67 - 1.72] | 0.754335 |
|  | GSE17710 | RFS | 56 | 0.94 [0.64 - 1.38] | 0.75172 |
|  | GSE17710 | OS | 56 | 1.05 [0.79 - 1.39] | 0.740389 |
| Ovarian cancer | GSE9891 |  | 278 | 1.27 [1.09 - 1.49] | 0.00213467 |
|  | GSE9891 |  | 278 | 1.13 [1.01 - 1.27] | 0.037494 |
|  | GSE9891 |  | 278 | 1.12 [0.93 - 1.36] | 0.229939 |
|  | DUKE-OC |  | 133 | 0.97 [0.86 - 1.09] | 0.611378 |
|  | DUKE-OC |  | 133 | 1.03 [0.85 - 1.24] | 0.797615 |
|  | GSE8841 |  | 81 | 1.09 [0.71 - 1.69] | 0.695551 |
|  | GSE26712 |  | 185 | 1.32 [0.95 - 1.84] | 0.103125 |
|  | GSE26712 |  | 185 | 1.18 [1.00 - 1.39] | 0.0566037 |
|  | GSE26712 | DFS | 185 | 1.39 [1.03 - 1.87] | 0.0301295 |
|  | GSE26712 |  | 185 | 1.18 [1.01 - 1.38] | 0.0378297 |
|  | GSE17260 | OS | 110 | 1.01 [0.86 - 1.19] | 0.891009 |
|  | GSE17260 | PFS | 110 | 1.06 [0.93 - 1.20] | 0.39708 |
|  | GSE14764 | OS | 80 | 1.30 [0.86 - 1.98] | 0.219717 |
|  | GSE14764 |  | 80 | 1.48 [0.92 - 2.37] | 0.103899 |
| Skin cancer | GSE19234 |  | 38 | 1.50 [0.74 - 3.07] | 0.263219 |
|  | GSE19234 |  | 38 | 1.43 [0.89 - 2.27] | 0.137399 |
|  | GSE19234 |  | 38 | 1.78 [1.12 - 2.83] | 0.0144658 |
| Soft tissue cancer | GSE30929 | DRFS | 140 | 1.32 [0.96 - 1.81] | 0.0897804 |
|  | GSE30929 |  | 140 | 1.35 [1.08 - 1.68] | 0.00757747 |

OS, Overall Survival; DFS, Disease Free Survival; DSS, Disease Specific Survival; EFS, Event Free Survival; RFS, Relapse Free Survival, DMFS, Distant Metastasis Free Survival; PFS, Progression Free Survival; DRFS, Distant Recurrence Free Survival; DSS, Disease Specific Survival; DMFS, Distant Metastasis Free Survival.
